# Contrasting Effects of Forest Type and Stand Age on Soil Microbial Activities: An Analysis of Local Scale Variability

**DOI:** 10.3390/biology10090850

**Published:** 2021-08-31

**Authors:** Anna Walkiewicz, Andrzej Bieganowski, Adrianna Rafalska, Mohammad I. Khalil, Bruce Osborne

**Affiliations:** 1 Institute of Agrophysics, Polish Academy of Sciences, Doświadczalna 4, 20-290 Lublin, Poland; a.bieganowski@ipan.lublin.pl (A.B.); adrafalska@ipan.lublin.pl (A.R.); 2 UCD School of Biology and Environmental Science and UCD Earth Institute, University College Dublin, Belfield, 4 Dublin, Ireland; i.khalil@prudencecollege.ie (M.I.K.); bruce.osborne@ucd.ie (B.O.); 3 School of Applied Sciences and Technology, Prudence College Dublin, 22 Dublin, Ireland

**Keywords:** forest soil, microbiology, enzymes, C cycle, basal respiration, soil microbial biomass, dehydrogenase, GHGs, C sequestration

## Abstract

**Simple Summary:**

The potentially important role of forests in climate change mitigation suggests a strong need for a more detailed understanding of these ecosystems. Besides climatic conditions, diverse forest vegetation creates varied conditions for the activity of soil microorganisms, and particular attention should be focused on a comprehensive study on the influence of different forest types on microbial activities. We conducted an experiment on six different forest soils (two coniferous, two deciduous, and two mixed sites comprising trees of different ages) collected from the same region (Lublin Upland, Poland) to assess the relationship between forest type and seasonal changes in microbial parameters. The annual mean values of the soil microbial indicators suggest that the mature deciduous stand was the most sustainable in microbial activities among the forest soils investigated. The diversity of the forest environment and the multifactorial dependence of the microbiological activity of forest soils necessitates further research in this field, especially using the same soil types. An understanding of forest ecosystem functioning can also be useful for forest management.

**Abstract:**

Understanding the functioning of different forest ecosystems is important due to their key role in strategies for climate change mitigation, especially through soil C sequestration. In controlled laboratory conditions, we conducted a preliminary study on six different forest soils (two coniferous, two deciduous, and two mixed sites comprising trees of different ages) collected from the same region. The aim was to explore any differences and assess seasonal changes in soil microbial parameters (basal respiration BR, microbial biomass C_mic_, metabolic quotient qCO_2_, dehydrogenase activity DHA, and C_mic_:C_org_ ratio). Indicator- and forest-specific seasonality was assessed. In addition to litter input, soil parameters (pH, nutrient content, texture and moisture) strongly regulated the analyzed microbial indicators. PCA analysis indicated similarity between mature mixed and deciduous forests. Among annual mean values, high C_mic_ and DHA with simultaneously low qCO_2_ suggest that the mature deciduous stand was the most sustainable in microbial activities among the investigated forest soils. Research on the interrelationship between soil parameters and forest types with different tree ages needs to be continued and extended to analyze a greater number of forest and soil types.

## 1. Introduction

An understanding of the microbial activity in forest soils is important given its role in the maintenance of the biodiversity of these ecosystems whilst also contributing to carbon (C) storage and greenhouse gas (CO_2_, CH_4_, N_2_O) fluxes [1,2,3]. Consequently, forests with appropriate management practices are often an important part of global biodiversity maintenance and underpin many strategies for climate change mitigation. Globally, forests cover 31% of all land surface [2], including 35% of the area of Europe, with a coverage of about 227 million ha in Europe [4]. Considering their types, coniferous stands dominate in Europe (46%), followed by broadleaved (37%) and mixed (17%) stands. In terms of the age of European forests, sites between 20 and 80 years dominate (accounting for 40%), while 12% and 18% represent sites below 20 years and over 80 years, respectively [5]. Forests grow in a range of climatic conditions and soil types [6], which is associated with various levels of biodiversity [2].

The soil ecosystem determines the growth environment for plants and forms a reservoir of available water and nutrients for trees, whose roots connect with soil microbial communities [7,8]. The activity of soil microbes reflects the functioning and condition of the forest ecosystem, since soil microorganisms are highly sensitive to changes and react faster to disturbances than many multicellular organisms [9,10]. Soil quality can be assessed using various microbial indicators [11]. Basal respiration (BR) is one of the oldest parameters, reflecting the biological activity of soil microorganisms and C availability for microbial growth. This includes the catabolic processes of aerobic microorganisms associated with organic matter decomposition and nutrient release or immobilization in the soil [1,10,12,13]. Soil microbial biomass (C_mic_) is an important ecological indicator of soil fertility and quality [14] and refers to the soil fraction that is responsible for the regulation of the transformation of organic matter and the cycling of nutrients and energy [10]. The metabolic quotient (qCO_2_) is a sensitive indicator of the likely constraints on the soil microbial community and can be used to assess ecosystem development and soil disturbance [15,16]. A low qCO_2_ value indicates that a greater part of C is built into the biomass and a smaller part is emitted as CO_2_. Among the soil microbial enzymes, the activity of intracellular dehydrogenase (DHA) is a bio-indicator of soil quality, health, and degradation and provides information about overall microbial activity in soils, because the enzyme is linked to viable cells [10,17,18,19]. Soil dehydrogenases act as carriers of electrons and hydrogen from the substrate to the acceptors during the early stages of soil organic matter oxidation; thus, the activity of these enzymes is also considered an indicator of oxidative metabolism in soils [18,19]. The C_mic_:C_org_ ratio can be used as a measure of the deterioration of soil quality [20], which determines the availability of soil C. In turn, C is necessary for growth and microbial activity [16] and is closely associated with the capacity of the soil to support populations of microorganisms [21]. Investigations that take into account this set of contrasting parameters allow a comprehensive assessment of soil (in our case—forest soil) quality.

Soil microbial biomass and activity in soils of different forest types show seasonal dynamics and are regulated by changes in climate, soil chemical and physical properties—mainly temperature, moisture, texture, pH, and nutrient availability [19,20,22,23,24,25,26,27,28,29,30]. The seasonality of BR may be highly dependent on the dynamics of soil temperature variability [31,32,33]. High microbial biomass was reported in pine forests in spring [34] and in a fir stand in summer [35]. A high C_mic_ in autumn may be an effect of a large amount of litter, which provides a source of nutrients and energy for soil microorganisms [36]. The metabolic quotient (qCO_2_) was higher in a cedar forest during the winter season and lower in a beech forest in the spring season [37], which demonstrates that changes in C use efficiency can occur during the year. Soil DHA is stimulated by a rise in temperature [29], with the decomposition of litter [38] reaching its lowest values in winter. Among the different physical parameters, soil texture influences microorganisms, since it regulates hydrology, aeration, and soil organic matter. Clay soils have a higher C_mic_ and DHA than sandy soils [7,23,26,27,39].

A specific aspect of the forest ecosystem is the regulation of soil microbial activity by trees of different ages representing different species, which produce diverse litters and root exudates and thus change soil hydrological and physical properties [40,41,42,43,44,45,46,47]. The effect of trees on soil microbial communities was observed in unfavorable site conditions with a low nutrient content [8]. It was reported that site conditions and tree species are very important factors defining the structure of the soil microbial community [48]. As well as the underlying soil type, differences in soil respiration may be an effect of the influence of different tree stands on the soil specific and on soil microorganisms [49]. Through the different ways that trees of different ages regulate the soil environment (pH, litter quality), this also results in changes in the enzymatic activity of soil [17,23,43]. Soil microbial parameters may also be significantly affected by nutrient (C, N, and P) content regulated by trees of different age; for instance, young trees need more N but less P than old trees [44,46]. Soil biota participation in C cycle results in their strong relationship with C sequestration and soil-atmosphere GHG exchange. The microbial contribution to C sequestration is governed by the interactions between microbial (the amount of biomass, the community structure, and microbial by-products) and soil properties (texture, clay mineralogy, and pore-size distribution) [50]. In addition to CO_2_ emission through the respiration process, the activity of different soil microorganisms, such as methanotrophs or nitrifiers, causes CH_4_ and N_2_O uptake.

To conduct preliminary research and better characterize the microbial populations associated with soils of diverse forests and vegetation, a comprehensive approach was taken in this study encompassing soils under different species and age of trees located in the same region (under similar weather conditions, Lublin Upland in Poland). We focused on a set of biochemical parameters related to microbial biomass/activity and C-use efficiency, that, in combination, allowed for a better interpretation of their role(s) in contrasting forest soils at the local scale. We hypothesized that interactions between climatic and soil parameters, forest type, and tree age significantly differentiate soil microbial indicators. We assumed higher values of soil microbiological biomass and activity and lower C use efficiency may be determined by:

(1) Higher temperatures in summer and spring;
(2) Higher water availability in spring than in autumn and winter (while drought conditions in summer may reduce microbial activity);
(3) Higher water storage and nutrient (N, P, C) availability in heavy vs. light soils;
(4) Lower water and higher nutrient demand of younger trees;
(5) The presence of leaf litter, which is most abundant in mature deciduous forests, and which could maintain a higher soil temperature in winter than in sites without litter and protect against excessive water loss in summer.

The aim of the study was to determine and utilize the microbial parameters: basal respiration, soil microbial biomass, metabolic quotient, dehydrogenase activity, and the C_mic_:C_org_ ratio for characterizing microbial activity and C use efficiency in six different forest soils in the same region (two coniferous, two deciduous, and two mixed sites) under trees of different age.

## 2. Materials and Methods

### 2.1. Site and Soil Characteristics

Soil samples were collected from six different forest sites located in the Lublin Upland, Poland. The basic characteristic of the forests examined are presented in Table 1.

**Figure 1:**
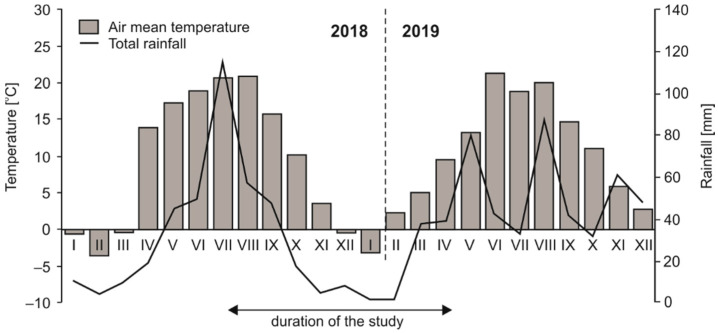
Meteorological parameters in the study region in 2018 and 2019, based on information from the weather station of the Institute of Agrophysics PAS located in Lublin.

During the sampling period (from July 2018 to April 2019), the annual mean temperature in the study area was 8.5 °C and the total annual rainfall was 330 mm. In the studied region, the highest mean temperature reached 20.7 °C in summer, while the lowest value (−0.4 °C) was recorded in winter. Based on the regional long-term data (2013–2018), the mean air temperature was 8.5 °C and mean total precipitation was 556 mm. Data for individual months are shown in Figure 1. The average temperature of the soils in situ were: 19.3 °C in summer, 9.9 °C in autumn, 2.5 °C in winter and 9.5 °C in spring.

Basic soil properties (Table 2) were determined for samples collected from a depth of 0–15 cm (*n* = 3) in July 2018 before the main experiment.

Soil texture was measured by the laser diffractometer Mastersizer 2000 with the Hydro G dispersion unit (Malvern Ltd., Malvern, UK). The following settings were used: two light sources of laser (633 nm) and diode (466 nm), stirrer speed 700 rpm, and pump speed 1750 rpm, soil refractive index 1.52 and soil absorption index 0.1 [51]. Soil pH was determined potentiometrically at room temperature in a soil and water slurry, after the soil had settled, with a ratio of soil:water of 1:2.5 *w*/*w*. The total C and N contents were determined in soils ground in a mortar using a dry combustion method (an oven temperature of 1020 °C; a Thermo Scientific Flash 2000 Organic Elemental Analyzer). Soil inorganic (SIC) and organic carbon (SOC) contents were determined with a TOC-VCPH analyzer (Shimadzu, Kyoto, Japan). Phosphorus (total P) concentration was determined by ICP-OES (Inductively Coupled Plasma Optical Emission Spectrometry) using a Thermo Scientific iCAP Series 6500 equipped with a charge injection device (CID) detector [52]. Soil temperature was measured during sampling using a TDR soil multimeter FOM/mts (*n* = 8). Undisturbed soil cores (100 cm^3^ brass cylinders) were tested after equilibration on a Richard chamber (Soilmoisture Equipment Corp., USA) in order to determine soil bulk density as the mass of a dry sample divided by the volume of the sample.

### 2.2. Experimental Design

The study was conducted on soil samples collected at monthly intervals in summer 2018 (July, August), autumn 2018 (September, October, November), winter 2018/2019 (January, February), and spring 2019 (April). The samples (surface layer, i.e., 0–15 cm depth after removal of litter) were collected at five randomly selected sites in each forest site, mixed, and thoroughly homogenized, and combined into one representative sample for the season and for each forest type. Representative samples were sieved through a 2 mm mesh, frozen (−20 °C) and next stored in the dark at 4 °C for four weeks before the microbial analyses to minimize microbial activity. To represent field conditions, the analyses were conducted at soil moisture levels corresponding to the natural conditions at the time of sampling (Table A1). The measurement procedures were performed after a two-day preincubation to stimulate microorganism activity (5 g of air-dry soils was placed in 60 cm^3^ glass vessels and incubated in the dark at 25 °C).

### 2.3. Analysis of Microbial Parameters

Soil basal respiration (BR, expressed in µg CO_2_-C/g/h) was measured after 2 h incubation at 25 °C. Soil microbial biomass (C_mic_) was determined using the substrate-induced respiration (SIR) method. Emission of CO_2_ was measured after enrichment of the soil with a glucose solution (10 mg per gram of soil) that provided an easily available source of carbon and energy [53]. After 2 h incubation with shaking at 25 °C in a water bath, the CO_2_ produced was collected by injection and measured chromatographically using a Shimadzu GC-14A (Shimadzu Corp., Kyoto, Japan). Microbial biomass content was recalculated according to Šimek and Kalčík, 1998 [54]. The activity of soil dehydrogenases (DHA) was determined with the Casida et al., 1964 [55] method using triphenyl tetrazolium chloride (TTC); this analysis was based on the amount of triphenyl formazan (TPF) produced after a 20 h incubation of the soil samples at 30 °C. After filtration, DHA (mg TPF/g/20 h) was measured spectrophotometrically at an absorbance of 485 nm (UV-1601PC, Shimadzu Corp., Kyoto, Japan). In total, 216 soil samples were used in this study to assess the microbial indicators. Based on the measured parameters, the C_mic_:C_org_ ratio and BR:C_mic_ ratio (the metabolic quotient qCO_2_ expressed as µg CO_2_-C/mg C_mic_/h) were calculated [56].

### 2.4. Calculation and Statistical Analysis

The values of the microbiological parameters were calculated for each season and each forest type. Based on the seasonal indicators, the average annual values of BR, C_mic_, qCO_2_, DHA, and C_mic_:C_org_ were calculated for each study site. The value of BR and qCO_2_ was corrected for CO_2_ dissolution in soil solution according to the Henderson–Hasselbach equation.

The results were statistically analyzed with Statistica 13 software (StatSoft Inc., Tulsa, OK, USA). A non-parametric Kruskall–Wallis test was used to evaluate the significance (at the 5% level) of the differences in soil parameters between the seasons in each forest and between the annual values for each forest separately. Principal component analyses (PCA) were carried out to examine the relationships among soil microbial indicators (BR, C_mic_, qCO_2_, DHA, C_mic_:C_org_), tree age, and soil parameters (sand, silt, and clay contents, moisture, pH, N, C, and P concentration, C:N, N:P, and C:P).

## 3. Results

**Figure 2:**
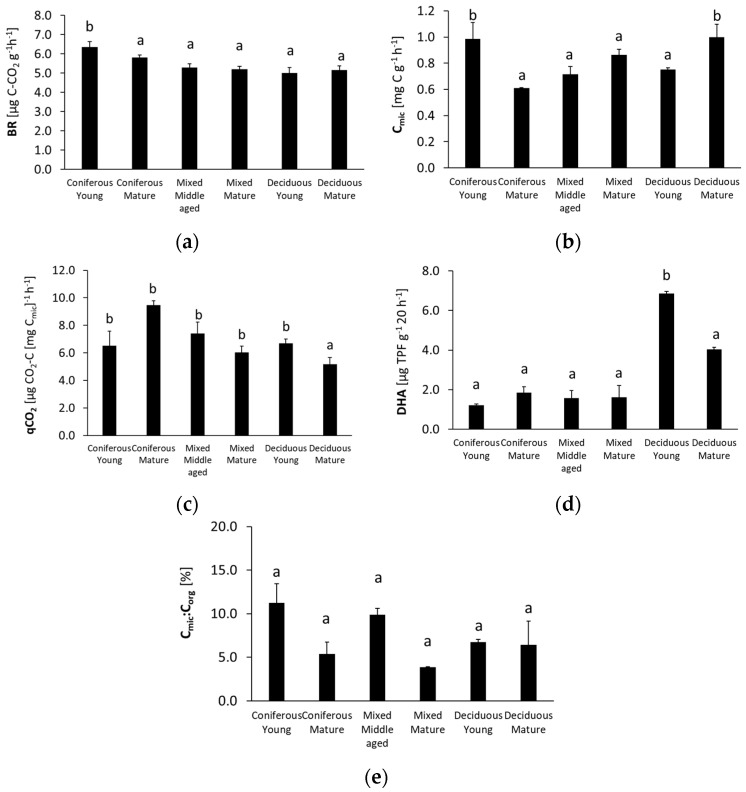
Annual mean microbial parameters of soils: (**a**) BR—basal respiration; (**b**) C_mic_—microbial biomass; (**c**) qCO_2_—metabolic quotient; (**d**) DHA—dehydrogenase activity, (**e**) and C_mic_:C_org_ ratio) collected from different forests (average values ± standard deviation). Different letters indicate significant differences (a non-parametric Kruskal–Wallis test, *p* < 0.05, was carried out separately for each parameter and for each forest).

**Figure 3:**
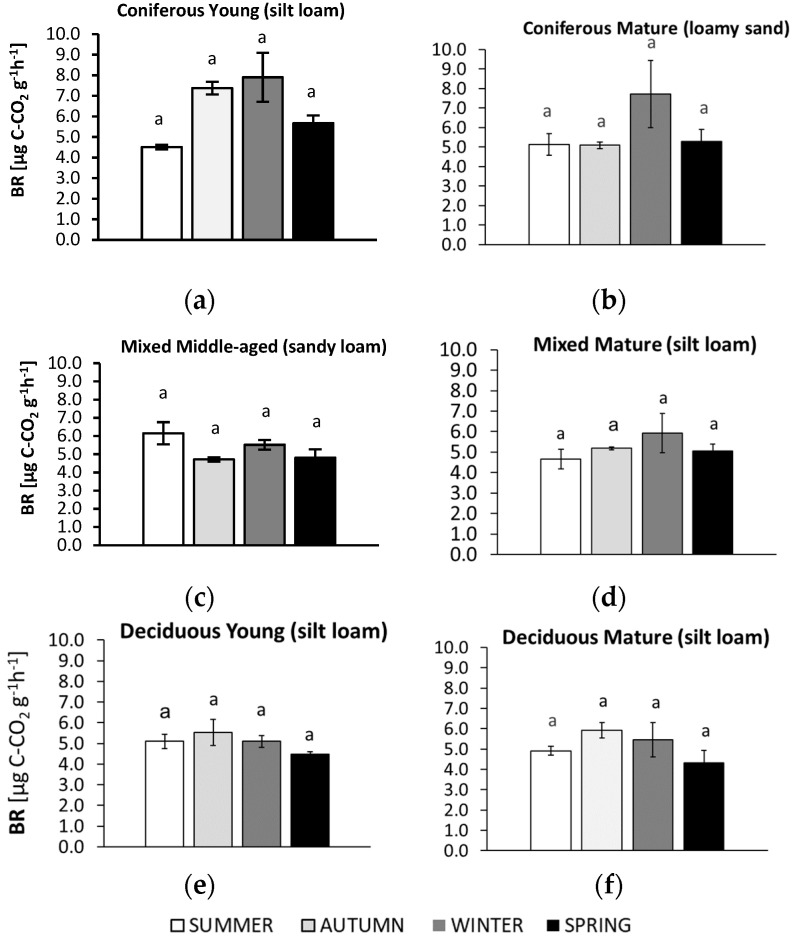
Basal respiration (BR) in soil samples collected in summer, autumn, winter, and spring from different forest sites (average values ± standard deviation; *n* = 3). (**a**) Coniferous Young (silt loam); (**b**) Coniferous Mature (loam sand); (**c**) Mixed Middle-aged (sandy loam); (**d**) Mixed Mature (silt loam); (**e**) Deciduous Young (silt loam); (**f**) Deciduous Mature (silt loam). Different letters indicate significant differences (a non-parametric Kruskal–Wallis test, *p* < 0.05, was carried out separately for each season and for each forest).

**Figure 4:**
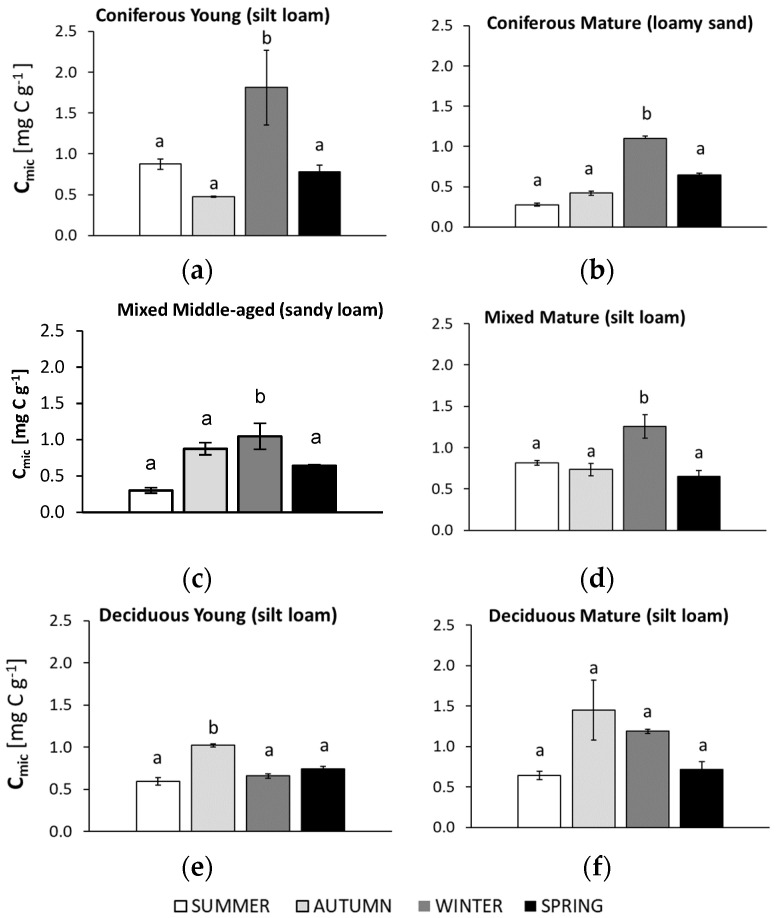
Soil microbial biomass (C_mic_) in samples collected in summer, autumn, winter, and spring from different forests (average values ± standard deviation; *n* = 3). (**a**) Coniferous Young (silt loam); (**b**) Coniferous Mature (loam sand); (**c**) Mixed Middle-aged (sandy loam); (**d**) Mixed Mature (silt loam); (**e**) Deciduous Young (silt loam); (**f**) Deciduous Mature (silt loam). Different letters indicate significant differences (a non-parametric Kruskal–Wallis test, *p* < 0.05, was carried out separately for each season and for each forest).

**Figure 5:**
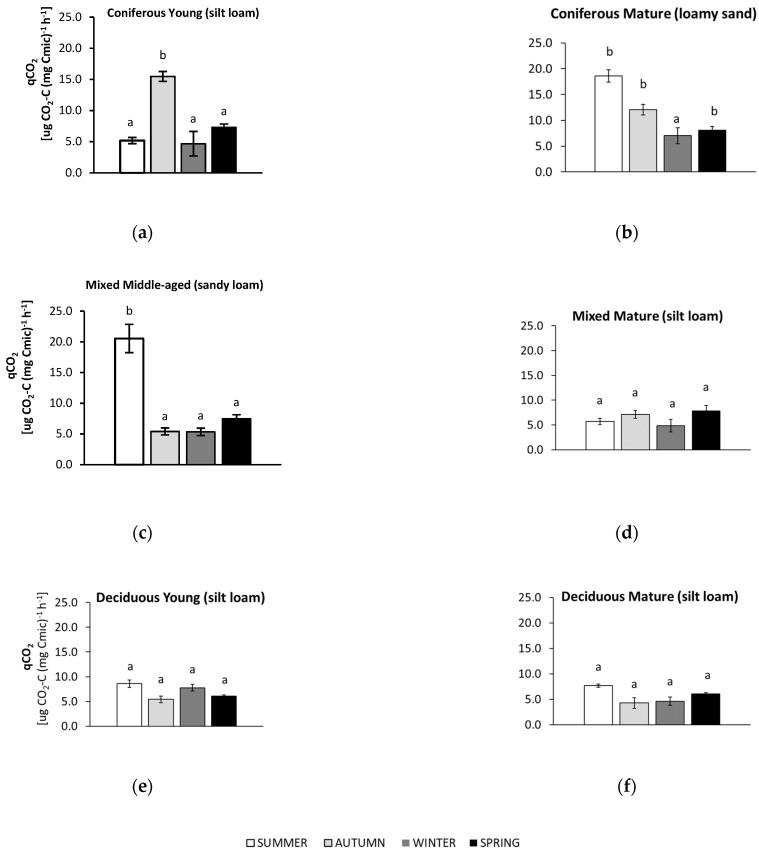
The metabolic quotient (qCO_2_) in soil samples collected in summer, autumn, winter, and spring from different forests (average values ± standard deviation; *n* = 3). (**a**) Coniferous Young (silt loam); (**b**) Coniferous Mature (loam sand); (**c**) Mixed Middle-aged (sandy loam); (**d**) Mixed Mature (silt loam); (**e**) Deciduous Young (silt loam); (**f**) Deciduous Mature (silt loam). Different letters indicate significant differences (a non-parametric Kruskal–Wallis test, *p* < 0.05, was carried out separately for each season and for each forest).

**Figure 6:**
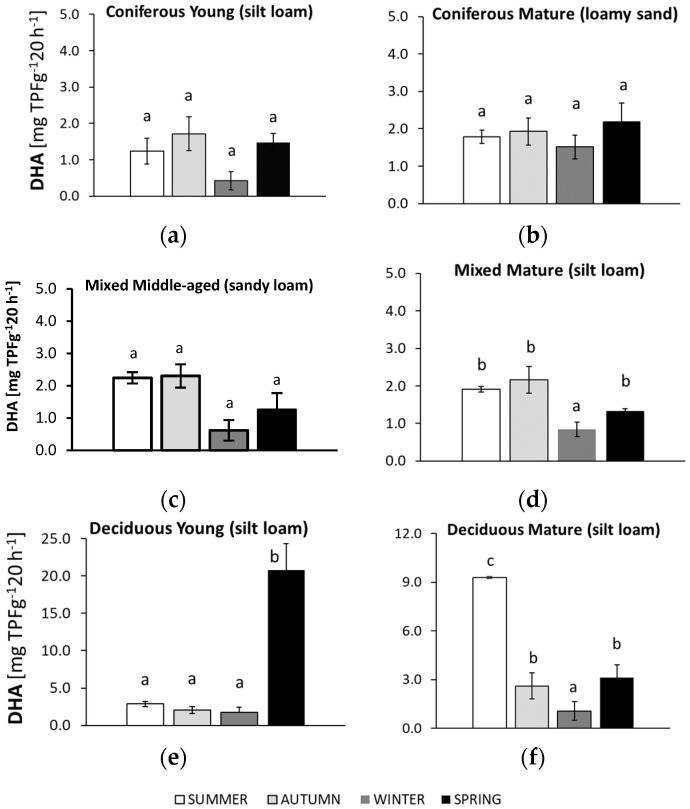
Dehydrogenase activity (DHA) in soil samples collected in summer, autumn, winter, and spring from different forests (average values ± standard deviation; *n* = 3). (**a**) Coniferous Young (silt loam); (**b**) Coniferous Mature (loam sand); (**c**) Mixed Middle-aged (sandy loam); (**d**) Mixed Mature (silt loam); (**e**) Deciduous Young (silt loam); (**f**) Deciduous Mature (silt loam). Different letters indicate significant differences (a non-parametric Kruskal–Wallis test, *p* < 0.05, was carried out separately for each season and for each forest).

**Figure 7:**
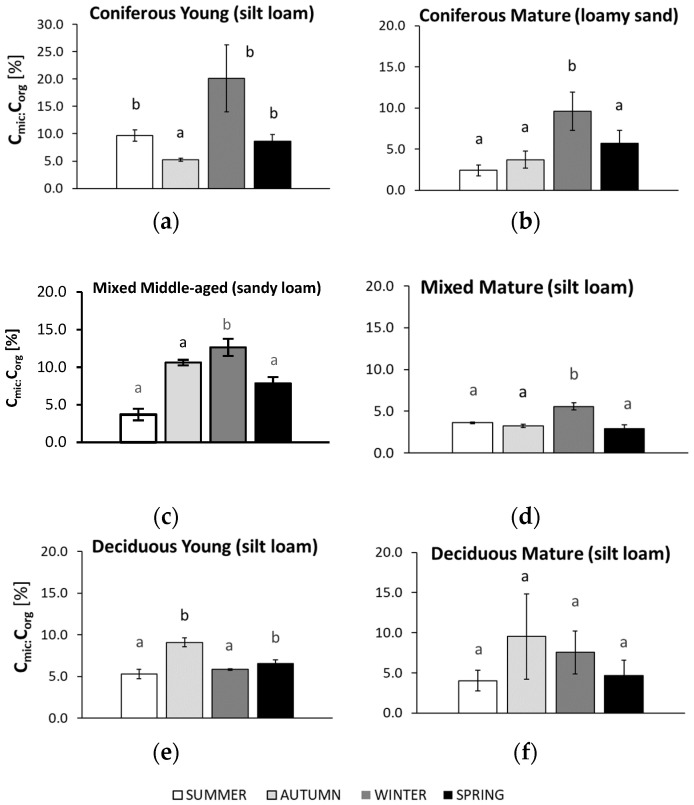
C_mic_:C_org_ ratio in soil samples collected in summer, autumn, winter, and spring from different forests (average values ± standard deviation; *n* = 3). (**a**) Coniferous Young (silt loam); (**b**) Coniferous Mature (loam sand); (**c**) Mixed Middle-aged (sandy loam); (**d**) Mixed Mature (silt loam); (**e**) Deciduous Young (silt loam); (**f**) Deciduous Mature (silt loam). Different letters indicate significant differences (a non-parametric Kruskal–Wallis test, *p* < 0.05, was carried out separately for each season and for each forest).

The studied microbiological parameters of the forest soils showed different annual mean values (Figure 2). The preliminary results of seasonal variability of the tested microbial parameters are shown in Figure 3, Figure 4, Figure 5, Figure 6 and Figure 7.

### 3.1. Annual Mean of Microbiological Parameters of Forest Soils

The annual mean values of each analyzed microbiological parameter are shown in Figure 2.

The highest BR was recorded in the soil from the young coniferous forests. It was about 20% lower for the rest of the studied forest soils. The mean annual C_mic_ had significantly higher values for the silty soils collected from the young coniferous forest and from the mature deciduous forest. It was about 40% lower for the sandy soil and the mature coniferous forest. The mean qCO_2_ value in silty soil from deciduous mature forest was significantly lower than in other stands, and the highest values were found in the sandy soils from the mature coniferous forest. The annual soil DHA values decreased in the following order: young deciduous > mature deciduous > mature and young coniferous and mixed. The highest annual C_mic_:C_org_ ratio was found for the silty soil of the young coniferous and middle-aged mixed forest, and values for these mature forests were approximately 50% lower.

### 3.2. Seasonal Changes in Soil Basal Respiration (BR)

Seasonal changes in BR were observed in all studied forest soils (Figure 3), although the pattern varied and differences between seasons were not significant.

In the mature coniferous forest on the sandy soil, BR was about 40% higher in winter compared to the other seasons. A higher BR in winter was also observed in the young coniferous forest on the silty soil; a similar level of BR was observed in autumn, but lower values were recorded in summer and spring. For the sandy soil from the middle-aged mixed forest, a higher BR was recorded in samples collected in summer; BR was about 24% lower in the soil collected in autumn and spring. In samples from the mature mixed forest on silty soil, the highest BR was found in winter and the lowest in summer. Silty soils of both deciduous forests had the highest BR value in autumn and the lowest value in spring, which did not differ significantly from the level observed in summer and winter.

### 3.3. Seasonal Changes in Microbial Biomass (C_mic_)

The seasonal changes in microbial biomass (C_mic_) are shown in Figure 4.

In all soils collected from the coniferous and mixed forests, the highest soil microbial biomass (C_mic_) was observed in winter. The young coniferous forest soil was the only case where the lowest C_mic_ was found in autumn and was almost two-fold lower than in samples collected in summer, autumn, and winter. The sandy (from mature coniferous and middle-aged mixed stands) and silty soils (from deciduous forests) showed the lowest C_mic_ in summer. The lowest C_mic_ in the silty soil from the mature mixed forest was recorded in spring, although this value did not differ significantly from the results obtained in samples from summer and autumn. The highest C_mic_ was recorded in samples from autumn only in the soils from the deciduous forests. Inconsistency due to the large standard deviation should be considered in the case of young coniferous and mature deciduous stands.

### 3.4. Seasonal Changes in Soil Metabolic Quotient (qCO_2_)

The results of the metabolic quotient (qCO_2_) are presented in Figure 5.

In summer, soils from most forests (mature coniferous, mature and young deciduous, middle-aged mixed) showed the highest metabolic quotient (qCO_2_). For the sandy soil collected from the mature coniferous forest, qCO_2_ was also high in autumn, while similar and significantly lower values were found in winter. For the young coniferous forest, the highest qCO_2_ was recorded in the autumn, and the values for the other seasons were approximately 3 times lower. The soils from the deciduous forests and the mature mixed forest had the lowest qCO_2_ values throughout all seasons. The lowest qCO_2_ values were noted in the silty soils collected from these forests in autumn (both deciduous forests), winter (mature deciduous and mature mixed site), and spring (young deciduous site). The greatest (approximately 4-fold) difference in qCO_2_ values between the highest (in summer) and lowest (in the other seasons) was recorded in the sandy soil from the middle-aged mixed forest.

### 3.5. Seasonal Changes in Soil Dehydrogenase Activity (DHA)

Significant seasonal changes in DHA occurred in all soils, apart from those collected from the mature coniferous forest (Figure 6).

Values for DHA in the sandy soil of the mature coniferous forest did not differ significantly across all seasons, although the highest numbers were observed in soil samples collected in spring. The silty soil from the young deciduous forest had particularly low values that were approximately 10 times higher in spring compared to the other seasons. In the soils from both mixed forests, the highest DHA was observed in samples from summer and autumn. The silty soil of the mature deciduous forest was the only soil that reached the highest DHA in summer. The value was three times higher than in the other seasons. The lowest DHA was recorded in the soils from winter in all the studied forests.

### 3.6. Seasonal Changes in Soil C_mic_:C_org_ Ratio

The seasonal changes in the C_mic_:C_org_ ratio in the forest soils are shown in Figure 7.

In soils collected from both coniferous forests, the highest C_mic_:C_org_ ratios were found for samples collected in winter, reaching values that were approximately two times greater than those from the younger forest. The lowest C_mic_:C_org_ values were found in the sandy soils of the mature coniferous forest collected in summer and in the silty soil of the younger coniferous forest collected in autumn. For the mature mixed forest, the highest C_mic_:C_org_ values were found in winter. For the sandy soil of the middle-aged mixed forest, the highest C_mic_:C_org_ ratios were recorded in autumn and winter and were about 3 times lower than those collected in the summer. The C_mic_:C_org_ ratio in the silty soil of the mature deciduous forest did not differ significantly across all seasons, but the highest value was noted in autumn and the lowest in summer and spring. Similarly, in the silty soil collected from the young deciduous forest, the highest C_mic_:C_org_ ratio was recorded in the autumn samples. It was about half that of the samples collected in summer.

### 3.7. Principal Component Analysis

**Figure 8:**
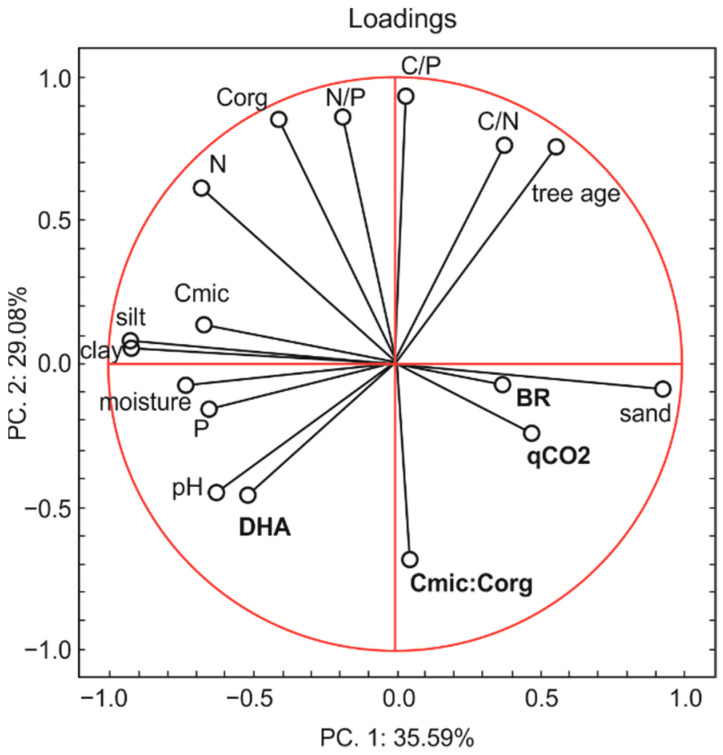
Principal component analysis (PCA)—biplot presentation between microbial and physicochemical parameters of six forest soils.

Principal component analysis (Figure 8), including a total of five microbial indicators (BR, C_mic_, DHA, qCO_2_, C_mic_:C_org_) and twelve parameters (tree age, sand, silt, and clay contents, pH, C, N, P content, moisture, C:P, N:P and C:N) in six forests, confirmed the high sensitivity of different microbial parameters to soil characteristics. The analysis generated two principal components. The first and second principal components (PC1 and PC2) explained 64.67% of the total variability of the data set. PC1 was negatively correlated with DHA, C_mic_, pH, P, moisture, silt and clay content, N, C_org_, and N/P and positively correlated with BR, qCO_2_, C_mic_:C_org_, sand content, tree age, C:N, and C:P. PC2 was negatively correlated with C_mic_:C_org_, DHA, qCO_2_, BR, sand content, pH, P, moisture and positively correlated with C_mic_, tree age, clay and silt content, N, C_org_, N:P, C:P, and C:N.

**Figure 9:**
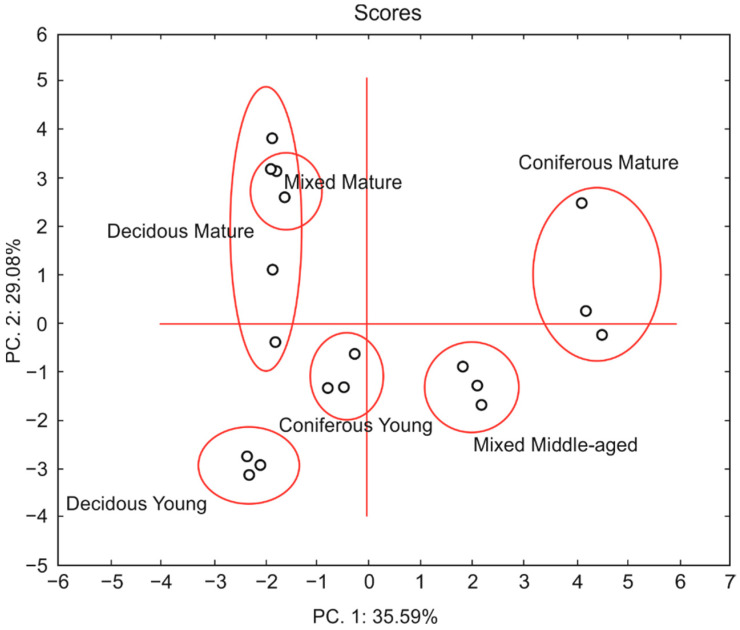
PCA plot of the properties of soils from mature and young coniferous, deciduous, and mixed forests.

The PCA analysis of forest soils clearly distinguished different forest types and tree age, although it failed to separate the mixed mature forest from the mature deciduous forest (Figure 9).

The mature coniferous and middle-aged mixed forests had positive PC1 scores, whereas the young coniferous and deciduous forests and the mature mixed and deciduous forests had negative PC1 scores. Clusters of samples from the mature forests had mostly positive PC2 scores, while those from the younger forests were found in the negative PC2 fields.

## 4. Discussion

The potentially important role that forests may have in climate change mitigation, especially through C sequestration, suggests the need to identify the variability and drivers of soil microbial activity in different forest types and stand ages. It is well-known that interactions among vegetation, climate and soil conditions have a combined effect on soil microbes. It was previously reported that forest type may affect soil microbial structure and activity through the presence of different tree species of different ages, and that this controls the soil biota [7,8,22,23]. Diverse stands also produce diverse litters, produce different amounts and type of root exudates, and influence soil hydrology and nutrient content [40,41,42,43,44,45,46,47]. As revealed by the PCA (Figure 9), different stands generally have contrasting impacts on soil microbiology. Interestingly, this analysis indicates that stand age is also an important factor regulating microbial activity. Although research on the same soils and different trees would show more precise differences, this preliminary study on forest soils from the same region (with similar weather conditions) presents results that are valuable and are important for understanding local scale variation.

### 4.1. Soil Basal Respiration (BR)

Seasonal differences in BR were most clearly visible in the soils collected from the coniferous forests but were not evident in the other forested sites. Unexpectedly, the highest BR was recorded in the winter samples (and in the autumn soil from the young forest), which may be the result of the thawing process on the growth and activity of soil bacteria [57,58]. However, these results should be interpreted taking into account the litter layer and the snow cover that protect the soils from complete freezing. The samples taken were stored at 4 °C before tests and analyzed in 25 °C according to standard procedures. Increasing temperatures may result in the enhanced growth of some bacteria, while during thawing, C and N can be released [59]. Low BR during spring (Figure 3) may be a result of quite low soil temperatures (about 10 °C) [31,32,33], while a high soil BR suggests the rapid decomposition of organic residues and the stimulation of heterotrophic microorganisms in coniferous forests.

A study on different soils in Germany reported that forest type had a significant effect on BR only in nutrient-poor sites, and BR in soils collected from a pure beech forest was 65% higher than in soils under Douglas fir [8]. In our study, soil collected from the young coniferous forest had a high mean annual BR (Figure 2). Malchair and Carnol (2009) [60] also showed higher BR values in soils under two spruce forests, which was explained by low substrate quality resulting in lower C use efficiency, as well as a low quality of litter or high organic matter content.

### 4.2. Soil Microbial Biomass (C_mic_)

**Figure A1:**
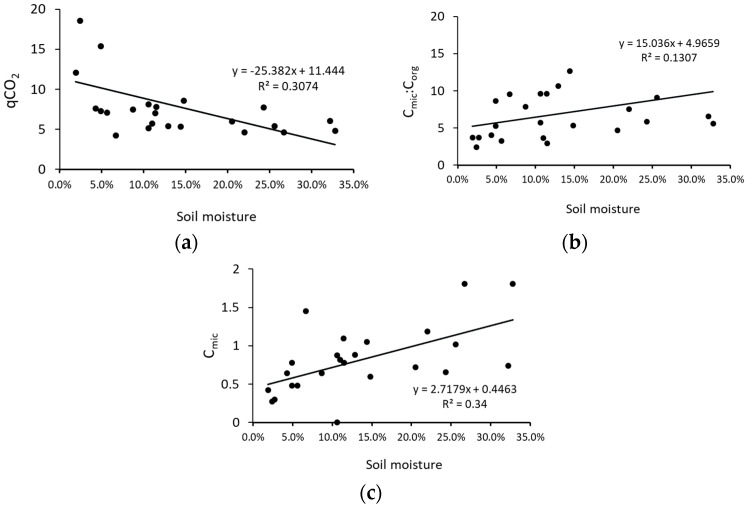
Correlations between soil moisture content and microbial parameters based on mean values for each season for all studied forests (*n* = 24) (**a**–**c**).

The seasonal dynamics of C_mic_ relates to changes in soil microbial biomass. The lowest C_mic_ values for most of the analyzed soils were found in the summer samples and were especially low in the soils of the mature coniferous and middle-aged mixed forests (Figure 4). This may be a result of low moisture combined with higher temperatures, which may limit the growth and activity of soil microorganisms [61,62]. A positive correlation between soil moisture and C_mic_ is shown in Figure A1. Although other studies have shown that the soil microbial biomass may be resistant to drought [62], the high metabolic quotient (qCO_2_) in the soil collected in the summer from the young mixed forest (Figure 5) may suggest a greater degree of stress than in other seasons in this stand. The highest C_mic_ values in the deciduous forests were recorded in the soils sampled in autumn (Figure 4); this may be related to the large amount of litter, which provides nutrients and energy for topsoil microorganisms during this season [36]. It is notable that the autumn C_mic_ in the soil under the mature deciduous forest was about three times higher than in the soil from both coniferous forests, which we assume was a consequence of the thicker litter layer in the deciduous site [34,63,64]. There was also an approximately three-fold larger amount of tree biomass in the mature deciduous forest compared to the coniferous forests (Table 1). Soil microbial biomass may also increase with the diversity of trees [65] and was high in the mature mixed forest analyzed in the present study (Figure 2).

In our study, the soils under the coniferous and mixed forests showed the highest C_mic_ values in winter, as they may have been protected and more active under litter and snow cover [66]. During freezing-thawing events that could occur locally, microbial cells are destroyed, and damaged cells release nutrients that can be used by the surviving microbes, which can be highly active during soil thawing [67]. A high microbial biomass in the winter season was also observed in pine and oak stands, which was explained by the larger proportion of fungi in the biomass [68,69,70]. Similar to our study, a lower C_mic_ was observed in summer than in winter, which may suggest that the water availability may have limited C_mic_ more than temperature [68,69].

The mean annual C_mic_ decreased in the following order: young coniferous forest > mature deciduous forest > mature mixed forest > young deciduous forest > middle-aged mixed forest > mature coniferous forest (Figure 2). Some authors suggest that the C_mic_ content is often higher in soils of older forests compared to younger-aged forests [71,72,73], which in our experiment was confirmed only in the soil of the deciduous and mixed stands. In contrast, the C_mic_ value in the coniferous forests was significantly higher in the soil of the young forest compared to the older one. Texture also regulates C_mic_, and in our study, silty soils showed a higher C_mic_ than the sandy soils (Figure 2). Texture influences microbial biomass since it affects the distribution of micro-, mezo- and macropores that create different microenvironments and determines water holding capacity, nutrient availability and oxygen concentration [7,26,27,39]. Soil with a higher clay fraction can maintain larger microbial communities due to the greater absorption and accumulation of organic C, and the maintenance of a higher water storage capacity [23,34,74,75].

### 4.3. Microbial Metabolic Quotient (qCO_2_)

The seasonality of the qCO_2_ value reflects variations in the bioenergetic status of microbial biomass [76] and the proportion of C_org_ that can be easily metabolized [77]. In soils from the mature coniferous forest and from the middle-aged mixed forest, the qCO_2_ value was almost four times higher in summer than in the other seasons (Figure 5). It is known that a higher qCO_2_ is a result of lower metabolically efficient microbial activity due to the effects of stress or ecosystem disturbances [15,78,79]. One of the unfavorable factors limiting the soil microbial activity may be summer droughts, which have been reported in European forests with increasing frequency [80,81,82,83]. In our study, the lower the moisture, the higher the qCO_2_ value that was observed (Figure A1). An explanation for the summer sensitivity of the qCO_2_ of the soils from the mature coniferous forest may be their sandy texture (Table 2), which store less water than silty soils. We also observed a higher qCO_2_ in the soils collected in autumn from both coniferous forests (Figure 5). Malchair & Carnol, (2009) [60] showed that, in September, the highest qCO_2_ was found in soils from a spruce forest compared with other forest types (beech, oak, Douglas fir) because of the poor litter quality of coniferous stands. Moreover, a higher qCO_2_ may also be linked with a lower C use efficiency in young stands [84,85] and a higher BR in a spruce forest [60].

The annual summary shows a clear relationship between the forest stand and the qCO_2_ value (Figure 2); qCO_2_ decreased in the following order: mature coniferous > middle aged mixed > young coniferous > young deciduous > mature mixed > mature deciduous. Almost a four-fold higher qCO_2_ was recorded in topsoil under coniferous compared to mixed forest in a study conducted in Italy by Agnelli et al. (2001) [86]. Higher seasonal changes (Figure 5) and higher annual qCO_2_ values (Figure 2) in soils from coniferous compared to deciduous stands may be a result of the presence of decomposition-resistant litter [87] associated with waxes, phenolic acids, and lignin contained in coniferous litter [88,89,90].

### 4.4. Soil Dehydrogenase Activity (DHA)

All preliminarily analyzed soils, except the sample collected from the mature coniferous forest, showed seasonal variations in DHA (but not always significant, Figure 6), reflecting changes in the overall activity of soil microorganisms. These differences may be associated with temperature changes. As reported by Wolińska & Stępniewska, (2012) [29], the highest DHA was observed at 20–30 °C, which may correspond to temperatures characteristic of summer and early autumn in the studied period (Figure 1). Moreover, this temperature range is comparable to the optimal value for the growth and activity of soil microorganisms [91]. DHA can also be stimulated by compounds released during litter decomposition [38], which is more intensive under wet conditions. Based on the meteorological data, we assume that the summer may have created near optimal conditions for DHA activity, since July 2018 was also distinguished by the highest rainfall (Figure 1). As a consequence, soil from the mature deciduous forest had the highest DHA value in this season (Figure 6), in contrast to the soil collected in the summer from the young deciduous stand where there was less aboveground biomass (Table 1) and a low litter level.

Tree species composition has been suggested as one of the main factors influencing soil DHA variations in forests [19], since the presence of vegetation may indirectly affect DHA by increasing fine root biomass in the soil [92] and influence litter amount and quality [93]. Mean annual DHA was significantly higher in the soils under deciduous forests and significantly lower in soils under the coniferous and mixed forests (Figure 2). With the increasing dominance of conifers, the conditions for organic matter decomposition were less favorable and associated with decreasing soil pH [17,23]. Soils under coniferous forests have an acidic pH, which may result in lower DHA compared to soils under deciduous forests [23]. The highest DHA was observed in the soil under the young deciduous forest (Figure 2), which was also characterized by the highest pH of all studied sites (Table 1). Similar to our results, a higher DHA in soils with a higher pH was confirmed in other studies on forest soils [22,23,28]. However, the impact of soil pH may be determined by the range of values to which the microbes are exposed. In our study, the soil pH varied from 4.25–5.37, while the optimum pH for DHA was reported to be about 7 [94,95,96].

### 4.5. Soil C_mic_:C_org_

The studied soils showed seasonal differences in C_mic_:C_org_, although the differences were not always significant between all seasons (Figure 7). High C_mic_:C_org_ indicates a favorable environment for microbial growth, whereas a low ratio may be related to poor quality of the soil organic matter [24]. In our study, higher qCO2 values were observed under higher soil moisture (Figure A1). In the summer, the C_mic_:C_org_ values were at similar levels in most soils, although higher values were found in the soil collected from the young coniferous forest. This may suggest that different forests may create similar soil conditions during the summer season. In our study, winter was the season with the highest C_mic_:C_org_ ratio in the coniferous and mixed forests, which could be a result of a higher soil moisture despite the lower temperatures [97].

The annual summary shows that the highest C_mic_:C_org_ in our study was found in the younger stands of the coniferous and mixed forests (Figure 2), which confirms that these stands created favorable conditions for microbial growth. Other studies have shown contrasting values for the soil C_mic_:C_org_ ratio in different forest types, with the lowest value in coniferous forests and the highest value in a deciduous forest [98] that reflects the contribution of microbial biomass to soil organic C [99].

**Figure 10:**
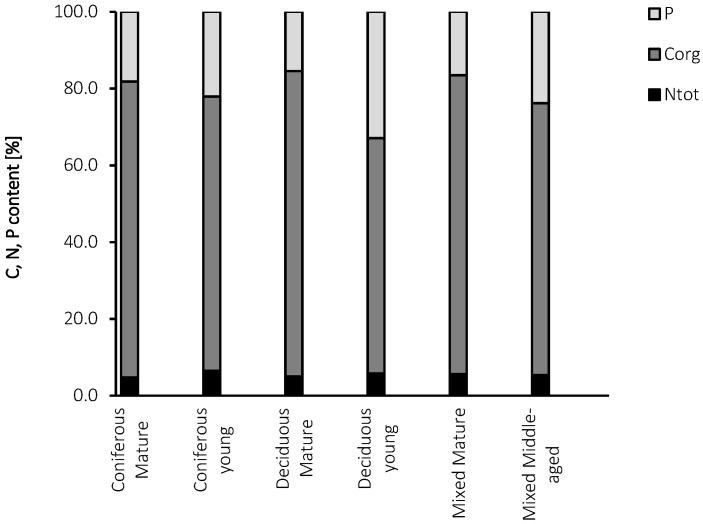
Contribution of C_org_, N_tot_, and P content in the analyzed forest soils.

Differences in the allocation of C, N, and P (Table 2, Figure 10) in the different forest types may also affect the microbial parameters. The uptake of C, N, and P normally constrains the growth of a range of organisms. Uptake from the environment is a key function, as these are the most important elements building living organisms [100], and the ratios of C, N, and P are key indicators of the nutritional status of soils [25].

Tree age may be also connected with soil nutrient ratios [40,41,42,44,45,46,47]. The contribution of N to nutrient content was lower in the younger forests in comparison to the mature stands (Table 2, Figure 10), which may be a consequence of the higher N demand of young compared to older trees [44]. An opposite trend is observed in the case of P, and soils associated with young forests may contain more P than old sites (Table 2, Figure 10), which may be related to the increase in P uptake with stand age [46]. European forest soils were reported to have C:N ratios below 20, with a mean C/N ratio of 16.5 and 15.3 for Cambisols and Luvisols, respectively [40]. These values were close to the mean C:N ratios in our soils (Table 2).

## 5. Conclusions

We concluded that the presented preliminary studies on forest soils from the same region showed seasonal and annual diversification of microbial parameters. Among the annual mean values, a high C_mic_ and DHA with a simultaneously low qCO_2_ suggests that the deciduous mature stand is the most sustainable in terms of microbial activities of the investigated forest soils. Based on the microbial parameters, we may predict that this ecosystem may be the most efficient in C use, considering the contribution of soil microbes in mitigating GHG emissions and enhancement of C sequestration, since it creates favorable conditions for microbiological activity. We revealed similarities between mature mixed and deciduous forests, which may indicate similar conditions for microbial activity. The soils may be more active in summer, autumn and spring than in winter, as confirmed by enzymatic (DHA) activity, as an effect of higher temperatures and litter, especially in soils from the mature deciduous forest and from both mixed forests. Since litter provides nutrients for soil microorganisms in the surface layer, we suggest that this may explain the highest C_mic_ in the soils collected in autumn from the deciduous forests. A low C_mic_ and high qCO_2_ were usually recorded in summer, which suggests that the conditions during this period limited the growth of soil microorganisms, probably due to low moisture. The results are valuable and are important for understanding local scale variation. The diversity of the forest environment and the multifactorial dependence of the microbiological activity of forest soils warrant further research in this area, especially in soil-tree interactions and their relationship with C sequestration and GHGs exchange.

## Figures and Tables

**Table 1:** Basic characteristic of the forests examined.

Forest Type	Coordinates	Tree Species	Contribution of Dominant Tree Species *	Tree Age *	Trees Biomass *
			[%]	[year]	[m^3^ ha^−1^]
Coniferous Mature	51.109675, 22.455327	Scot pine *Pinus sylvestris*	100	100	83
Coniferous Young	51.036295, 22.993784	Norway spruce *Picea abies*	100	36	68
Deciduous Mature	51.034831, 22.993789	Oak *Quercus robur* Aspen *Populus tremula*	70 30	71 63	193 87
Deciduous Young	51.217270, 22.670177	Sweet cherry *Prunus avium*	100	12	26
Mixed Mature	51.220832, 22.676460	Scot pine *Pinus sylvestris* Oak *Quercus robur*	80 20	85 85	306 65
Mixed Middle Aged	51.111152, 22.453757	Oak *Quercus robur* Scot pine *Pinus sylvestris* Black alder *Alnus glutinosa*	80 10 10	59 59 59	199 35 21

* Source: based on the Forest Data Bank, www.bdl.lasy.gov.pl (accessed on 30 August 2021).

**Table 2:** Properties of the different forest soils (0–15 cm depth; *n* = 3 avg. ± SD).

Forest Type	Coniferous Mature	Coniferous Young	Deciduous Mature	Deciduous Young	Mixed Mature	Mixed Middle Aged
Soil Type	*Dystric Cambisol*	*Albic Dystric Luvisol*	*Leptic Cambisol*	*Dystric Cambisol*	*Dystric Cambisol*	*Albic Luvisol*
Texture	loamy sand	silt loam	silt loam	silt loam	silt loam	sandy loam
Clay Content [%]	1.6 ± 0.11	5.6 ± 0.11	5.4 ± 0.13	4.8 ± 0.10	5.1 ± 0.15	2.0 ± 0.08
Silt Content [%]	22.9 ± 1.29	72.4 ± 0.35	69.7 ± 0.53	64.3 ± 1.40	69.4 ± 0.76	27.7 ± 0.82
Sand Content [%]	75.5 ± 1.40	22.1 ± 0.38	24.9 ± 0.62	30.8 ± 1.43	25.4 ± 0.66	70.3 ± 0.89
Bulk Density [g cm^−3^]	1.20	0.94	1.14	1.23	1.13	1.23
pH_H2O_	4.37 ± 0.006	4.82 ± 0.015	4.80 ± 0.006	5.34 ± 0.006	4.22 ± 0.021	4.79 ± 0.010
N [%]	0.074 ± 0.016	0.083 ± 0.004	0.108 ± 0.017	0.107 ± 0.002	0.163 ± 0.004	0.063 ± 0.001
C_org_ [%]	1.20 ± 0.363	0.91 ± 0.068	1.73 ± 0.670	1.14 ± 0.065	2.25 ± 0.103	0.82 ± 0.074
C/N	16.12 ± 1.39	11.04 ± 0.85	15.71 ± 3.68	10.66 ± 0.43	13.81 ± 0.30	13.17 ± 0.95
P_tot_ [mg/kg]	277.0 ± 6.74	273.6 ± 9.41	316.7 ± 23.81	456.8 ± 159.5	458.2 ± 20.36	264.2 ± 14.55
C/P	42.4 ± 12.94	32.3 ± 2.51	51.2 ± 18.03	18.6 ± 0.78	47.2 ± 2.29	29.7 ± 2.63
N/P	2.60 ± 0.56	2.93 ± 0.16	3.21 ± 0.46	1.77 ± 0.016	3.41 ± 0.16	2.25 ± 0.04

## Data Availability

The data presented in this study are available on request from the corresponding author. The data are not publicly available due to privacy.

## Appendix A

**Table A1:** Soil moisture content at the time of sampling tested soils [% *v*/*v*].

Forest Type	Summer	Autumn	Winter	Spring
Coniferous Mature	2.4%	1.9%	11.4%	10.6%
Coniferous Young	10.6%	4.9%	26.7%	4.9%
Deciduous Mature	4.3%	6.7%	22.0%	20.5%
Deciduous Young	14.8%	25.6%	24.3%	32.2%
Mixed Mature	11.0%	5.6%	32.8%	11.5%
Mixed Middle-aged	2.7%	12.9%	14.4%	8.7%
